# Transgenic mouse model for conditional expression of influenza hemagglutinin-tagged human *SLC20A1/PIT1*

**DOI:** 10.1371/journal.pone.0223052

**Published:** 2019-10-15

**Authors:** Sampada Chande, Bryan Ho, Jonathan Fetene, Clemens Bergwitz

**Affiliations:** Section of Endocrinology and Metabolism, Yale University School of Medicine, New Haven, CT, United States of America; University of Pittsburgh School of Medicine, UNITED STATES

## Abstract

To further investigate the role of the phosphate (Pi) transporter *PIT1* in Pi homeostasis and tissue mineralization, we developed a transgenic mouse expressing the C-terminal *influenza hemagglutinin (HA)* epitope-tagged human *PIT1* transporter under control of the cytomegalovirus/chicken beta actin/rabbit beta-globin gene (CAG) promotor and a loxP-stop-loxP (LSL) cassette permitting conditional activation of transgene expression (*LSL-HA-hPIT*^*tg/+*^). For an initial characterization of this conditional mouse model, germline excision of the LSL cassette was performed to induce expression of the transgene in all mouse tissues (*HA-hPIT1*^*tg/+*^). Recombination was confirmed using genomic DNA obtained from blood samples of these mice. Furthermore, expression of *HA-hPIT1* was found to be at least 10-fold above endogenous mouse *Pit1* in total RNA isolated from multiple tissues and from cultured primary calvaria osteoblasts (PCOB) estimated by semi-quantitative RT-PCR. Robust expression of the *HA-hPIT1* protein was also observed upon immunoblot analysis in most tissues and permits HA-mediated immunoprecipitation of the transporter. Characterization of the phenotype of *HA-hPIT1*^*tg/+*^ mice at 80 days of age when fed a standard chow (0.7% Pi and 1% calcium) showed elevated plasma Pi, but normal plasma iPTH, iFGF23, serum calcium, BUN, 1,25-dihydroxy vitamin D levels and urine Pi, calcium and protein excretion when compared to WT littermates. Likewise, no change in bone mineral density was observed upon uCT analysis of the distal femur obtained from these mice. In conclusion, heterozygous overexpression of *HA-hPIT1* is compatible with life and causes hyperphosphatemia while bone and mineral metabolism of these mice are otherwise normal.

## Introduction

Inorganic phosphate (Pi) has an essential role in cell signaling and metabolism and is tightly regulated by parathyroid hormone (PTH), 1,25-dihydroxy vitamin D (1,25-D), and fibroblast growth factor 23 (FGF23) to avoid excess or deficiency [1]. The type III transporters *PIT1* and *PIT2* have recently emerged as candidates for “transceptors” that mediate activation of ERK1/2 by Pi in a transport-independent fashion [2–4]. *PIT1* and *PIT2* mediate osteogenic differentiation of smooth vascular muscle [5], bone cells [6, 7], attenuation of ER-stress in chondrocytes [8], and insulin signaling [9], while pharmacological and genetic inhibition of *PIT1* and *PIT2* blocks these effects. Furthermore, *PIT1* and *PIT2* dimerization is stimulated by Pi independent of Pi transport [4].

Because of lack of suitable *in vitro* models, it remains unclear whether *PIT1* or *PIT2* regulate PTH, 1,25-D or FGF23. Ablation of *Pit1* in mice results in embryonic lethality at E12.5 [10, 11]. Hypomorphic *Pit1* ablation is viable and reduces femur length, but no significant changes of Pi or calcium metabolism were observed [12]. *Pit1* transgenic rats, on the other hand, have reduced trabecular number on uCT, hyperparathyroidism, and hyperphosphatemia, but FGF23 is not significantly different in these studies [13]. *Pit2* null mice have low bone mass [6] but normal Pi homeostasis at baseline and become hyperphosphatemic when fed a high Pi diet [3]. Along with inappropriately low intact FGF23 levels in these mice, these findings suggest that *Pit2* is upstream of and positively regulating FGF23. Based on these findings, it is possible that *PIT1* and *PIT2* compensate for each other preventing significant changes in the respective single gene ablation models.

To generate a mouse model that permits identification of *PIT1* binding partners that mediate activation of ERK1/2 downstream of Pi, for example by co-immunoprecipitation, we designed a transgenic approach for the conditional overexpression of an epitope-tagged version of this type III Pi transporter. To avoid unpredictable genomic events such as multiple insertions of plasmids, which are a common problem with standard transgenesis [14], and to prevent off-target recombination, which are a common problem with CRISPR/Cas9 technology [15], we decided here to use TARGATT technology to insert a C-terminally *influenza hemagglutinin (HA) -epitope tagged hPIT1* under a silenced CAG promotor into a modified *H11* locus [16].

This initial publication describes the phenotype of *HA-hPIT1*^*tg/+*^ mice which express the *HA-epitope tagged hPIT1* under the CAG promotor heterozygously after germline excision of the lox-stop-lox cassette. Different from observations in *Pit1* transgenic rats [13], *HA-hPIT1*^*tg/+*^ mice have, aside from hyperphosphatemia, normal bone and mineral metabolism.

## Materials and methods

### Animals

The research was approved Sept. 30, 2017, under IACUC protocol 2017–11635 by the Yale Institutional Animal Care and Use Committee (IACUC), valid through Sept. 30, 2020. Yale University has an approved Animal Welfare Assurance (#A3230-01) on file with the NIH Office of Laboratory Animal Welfare. The Assurance was approved May 5, 2015. Mice were weaned at 3 weeks of age and allowed free access to water and standard chow (1.0% calcium, 0.7% phosphate, of which 0.3% is readily available for absorption, Harlan Teklad TD.2018S). Genotyping was performed by PCR amplification of genomic DNA extracted from tail clippings or tail blood samples and amplified by polymerase chain reaction (PCR) as described [17]. Mice were euthanized at 80 days of age in deep anesthesia with isoflurane by retroorbital exsanguination and removal of vital organs.

#### Generation of germline transgenic HA-hPIT1^tg/+^ mice and genotyping by PCR at day P21

**Fig 1:**
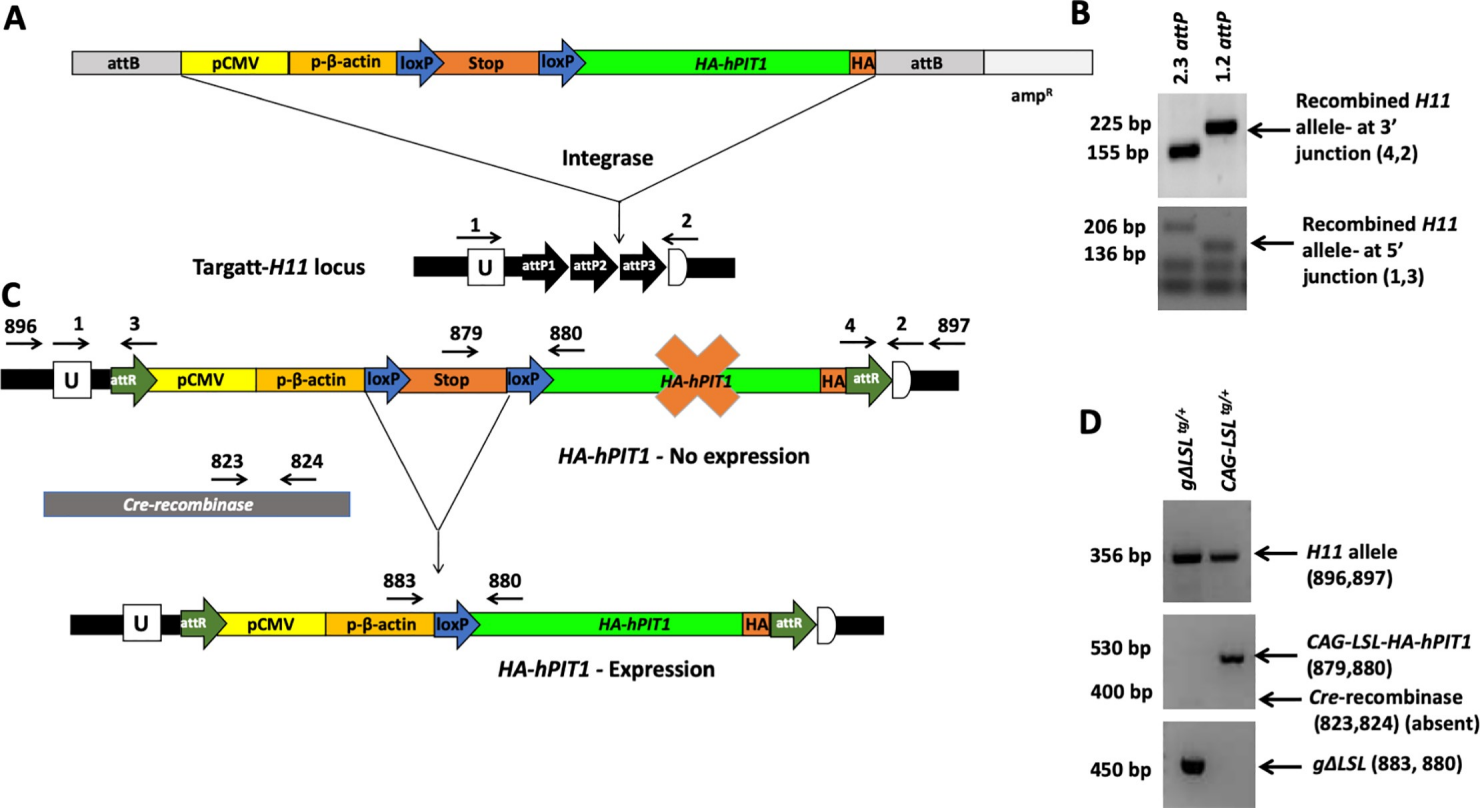
Generation of germline *HA-hPIT1*^*tg/+*^ transgenic mouse. Scheme for recombination of the targeting vector *pBT478*.*6-mKozak-CAG-LSL-HA-hPIT1 TARGATT* into the *H11* locus **(A)**. Primer positions are shown, which were used in (**B**) to detect the modified *H11attR* site at 5’ junction (transgene inserted between attP1 and attP2) and 3’ junction (transgene inserted between attP2 and attP3) using primers from the Applied StemCell kit (primers 1,3 and 2,4, respectively). Germline excision shown in (**C**) was confirmed in (**D**) using primers detecting the wildtype *H11* site (primers 896 and 897), the inactive *LSL*-positive (primers 879, 880) and the active germline *ΔLSL* alleles (primers 883 and 880). Primers 823 and 824 detect *Cre* recombinase, which is absent in the two mice shown consistent with germline excision of the LSL cassette of the mouse sample run in the left lane.

To generate pBT478.6-mKozak-CAG-LSL-HA-hPIT1, we used a PCR product which had been amplified with primer 816 containing the mouse Kozak sequence and primer 817 (see S1 Table for primer sequences) containing the influenza virus hemagglutinin (HA) sequence followed by a stop codon using a plasmid purchased from DFCI containing the full cDNA of human *PIT1* (HsCD00327712, Dana Faber, Boston, MA) as template. This C-terminal HA-tag was previously shown to be compatible with expression of human *PIT1* [18]. The resulting PCR product was inserted at the Avr-II/Mlu-I restriction sites into the multicloning region of *pBT378*.*6* (Applied StemCell, Inc., Milpitas, CA) downstream of the cytomegalovirus/chicken beta actin/rabbit beta-globin gene (CAG) promotor, which is silenced by a loxP-stop-loxP (LSL) cassette, permitting *Cre*-mediated activation of transgene expression (*CAG-LSL-HA-hPIT1*^*tg/+*^*)* (Fig 1A). 15 μg of endotoxin-free plasmid prep was further purified with the Qiagen PCR clean-up Gel extraction kit (Cat#: 740609), sequence verified and RNAse tested, and microinjected into fertilized oocytes harvested from hyperovulated *H11* females who had mated with *H11* males the previous night. The TARGATT transgenic kit (containing integrase mRNA) (Applied StemCell, Cat#: AST-1004) was used according to the manufacturer’s guidelines, with a minor modification to omit the kit’s supplement and used the Yale Genome Editing Center injection buffer miTE (0.1mM EDTA, 10mM Tris, pH 7.5). The resulting pups were genotyped using proprietary insertion site-specific primers 1–4 (Applied StemCell, Inc, Milpitas, CA), the *H11* primers 896 and 897 and *hPIT1*-specific primers 890 and 891 (see S1 Table) to confirm single copy insertion of *HA-hPIT1* into the *H11* locus. Using 8 kb-*Dmp1-Cre* mice (B6N.FVB-Tg(Dmp1-cre)1Jqfe/BwdJ Stock No: 023047 [19]) that permit transmission of *Cre* in the female germline, we facilitated excision of the *LSL* cassette and expression of the transgene in all mouse tissues (*HA-hPIT1*^*tg/+*^*)*. Successful recombination and absence of the *LSL* cassette was confirmed using primers 883 and 880, and 879 and 880, respectively (see S1 Table). Mice were analyzed at postnatal day (P) 80. The founder was outbred against C57Bl6 wild-type mice (*WT*) for at least 6 generations to obtain *HA-hPIT1*^*tg/+*.^and *WT* littermates to serve as controls with similar genetic background in our study.

### Biological chemistry

P80 mice were placed into fresh bedding to minimize coprophagy and fasted overnight (18 h) before collecting blood and urine. During overnight fasting, mice had free access to water. Biochemical analyses were done on blood samples collected after orbital exsanguination on the following day by 10AM. Concentrations of serum and urinary total calcium (Ca), blood urea nitrogen (S-BUN), and plasma and urine inorganic phosphorus (Pi) were determined using Stanbio Laboratories (Boerne, TX) kits #0155, #0580, #0830, respectively. The concentration of urine creatinine (U-crea) and serum 1,25-dihydroxyvitamin D (1,25(OH)_2_D) were determined using R&D systems (Minneapolis, MN) kits #KGE005 and #AC-62F1 at the Yale O’Brien Center and Yale Mineral Metabolism Lab, respectively. Concentrations of plasma intact parathyroid hormone (PTH) and intact fibroblast growth factor 23 (iFGF23) were determined using Immutopics/Quidel (San Clemente, CA) kits #60–2305 and #60–6800, respectively. Internal standards were used to assure reproducibility between batches. Fractional excretion indexes were calculated using the formula PEI = urine Pi/(urine creatinine*plasma Pi) or CEI = urine Ca/(urine creatinine*serum Ca), respectively.

### Histological analysis

Heart, aorta, brain, kidney, and lungs of P80 mice were fixed in 4% formalin/PBS at 4°C for 12 h and then dehydrated with increasing concentrations of ethanol and xylene, followed by paraffin embedding. Calcifications were determined on 10 um sections stained with von Kossa and 1% methyl green. Hematoxyline/eosin stained sections were used for morphological evaluation.

For transmission electron microscopy a 1 mm^3^ block of the left kidney was fixed in 2.5% Glutaraldehyde and 2% paraformaldehyde in phosphate buffered saline (PBS) for 2 hrs., followed by post-fixation in 1% osmium liquid for 2 hours. Following dehydration using a series of ethanol concentrations (50% to 100%), tissue was embedded in epoxy resin, and polymerization was carried out at 60°C for overnight. After preparing a thin section (50 nm), the tissues were double stained with uranium and lead and observed using a Tecnai Biotwin (LaB6, 80 kV) (FEI, Thermo Fisher, Hillsboro, OR) at the Yale Center for Cellular and Molecular Imaging (YCCMI).

### Micro computed tomography

The left leg of P80 mice was fixed in 70% ethanol at 4°C and analyzed using microCT-35 at the Yale uCT Facility (Scanco Medical AG, Brutisellen, Switzerland).

#### Primary calvaria-derived osteoblastic cell culture

Primary calvaria-derived osteoblastic cells (PCOB) were obtained from either WT or *HA-hPIT1*^*tg/+*^ mice at P80 as previously described [20]. Calvariae were dissected from adult mice, washed with PBS to remove soft tissue, chopped into 1–2 mm^3^ pieces, and digested with 2 mL collagenase type A, prepared as 300 active U/mL (Collagenase A, Roche, IN) dissolved in α-modified essential medium (α-MEM) (12571–063, Gibco, NY), for 30 minutes at 37°C, 200 rpm. The solution was discarded and replaced with fresh collagenase solution and incubated for 3 hours. The fragments were cultured in 6-well plates coated with type-I rat tail collagen (C3867-1VL,Sigma,MO) and grown in α-MEM supplemented with 5% fetal bovine serum (F4135, Sigma, MO), 5% calf serum (12133C, Sigma, MO), and Penicillin-Streptomycin (P4333, Sigma, MO). After 8–10 days, cells were split and transferred into 24-well plates for evaluation of mRNA and protein expression.

### Gene expression analysis by semi-quantitative RT–PCR

Total RNA from PCOBs and mouse tissues at P80 was prepared using RNeasy columns (Qiagen, Valencia, CA) or Trizol procedure (Life technologies, Carlsbad, CA), respectively, and transcribed into cDNA using the Omniscript kit (Qiagen, Valencia, CA). Semi-quantitative PCR was performed in an ABI-Step One Plus Cycler (Fisher, Life Technologies, Waltham, MA) using QuantiTect reagents (Qiagen, Valencia, CA) and mouse *beta actin*, mouse *Pit1*, and human *PIT1* primers (see S1 Table) and analyzed using the 2^-ΔΔCt^ method.

### Immunoprecipitation and immunoblot analysis

For immunoblot analysis, PCOBs or mouse tissues prepared from P80 mice were lysed in a buffer containing 62.5 mM Tris HCL (pH 6.8), 1% Nonidet P-40, 100 mM Na-orthovanadate, 2% beta mercaptoethanol, and 0.0015% bromophenol blue to prevent lysis of nuclei, and the cytosolic and cell membrane fraction was used for PAGE gel electrophoresis on 10% Tris-HCl/Glycine SDS-polyacrylamide (456–1034, BioRad, CA), electro-transferred to PVDF membranes (162–0218, BioRad, CA) and hybridized with anti-PIT1 rabbit polyclonal antibody (H-130, sc-98814, Santa Cruz Biotechnology) 1:1000 or anti-HA species/type (Y11, sc-805-G, Santa Cruz Biotechnology) in PBS containing 0.1% Tween 20 (PBST) and 5% non-fat dry milk at 4°C overnight, and detected with horseradish-peroxidase conjugated anti-rabbit IgG (7074S, Cell Signaling Technology, MA) in PBST+5% non-fat dry milk at room temperature for 60 min. using chemiluminescence/autoradiography (Supersignal 34580, Thermo Scientific, Waltham, MA). For immunoprecipitation, 7 mg tissue was homogenized in 500 uL of RIPA buffer (50 mM Tris HCl (pH 6.8), 150 mM NaCl, 1% Triton X-100, 0.5% sodium deoxycholate, 0.1% sodium dodecyl sulfate, Protease inhibitor without EDTA (Roche, Mannheim, Germany), 0.4 mM Na-orthovanadate, 50 mM beta-GP, and 5 mM NaF) using an electric homogenizer. Lysate was placed on an orbital shaker for 2 hrs. at 4°C and centrifuged to remove debris. Supernatant was diluted 10 times in buffer containing 50 mM Tris HCL (pH 6.8), Protease inhibitor without EDTA (Roche, Mannheim, Germany), 0.4 mM Na-orthovanadate, 50 mM beta-GP, and 5 mM NaF, and immunoprecipitated with anti-HA affinity matrix (Roche, Mannheim, Germany) at 4°C overnight. Matrix was washed 5 times in buffer containing 50 mM Tris HCl (pH 6.8), suspended in 2X Laemmli sample buffer (BioRad, Hercules, CA), and incubated on a shaker at 37°C for 60 minutes. Supernatant was loaded after pelleting the matrix. Densitometric analysis of Western blots was performed using ImageJ software (build: 269a0ad53f), and quantification was corrected for protein loading by normalization over the Coomassie stain.

### Statistical analysis

Data are expressed as means±SEM and were analyzed in Prism 8.0 (GraphPad Software, Inc., La Jolla, CA). Differences between groups were considered significant if p-values obtained with Student’s t-test were <0.05. The Mann-Whitney U test was used for comparisons when there was evidence by the Shapiro-Wilk normality test that the data were not normally distributed. Two-way ANOVA and Tukey’s test for multiple comparisons was used to determine significant differences between more than two treatment groups with significance threshold p<0.05.

## Results

### Generation of a transgenic mouse that expresses *influenza hemagglutinin (HA)-tagged human PIT1 (HA-hPIT1*^*tg/+*^*)*

To generate a transgenic mouse over-expressing an *HA-epitope tagged hPIT1 (HA-hPIT1)* under CAG promotor we used the plasmid *pBT378*.*6*, mice with a modified *H11* locus, and the TARGATT technique (Applied StemCell [16])(Fig 1A). We found integration of the expression cassette into the *H11* locus in one pup of the resulting litter (*CAG-LSL-HA-hPIT1*^*tg*^, Fig 1B), integration of the plasmid backbone in a second pup, and unexpectedly random integration in the remaining seven pups. The *H11*-*CAG-LSL-HA-hPIT1*^*tg*^ mouse line was crossed with *Dmp1-Cre*^*tg/+*^ mice that express cre-recombinase under the control of the 8 kb dentin matrix protein 1 promotor [19] to activate transgene expression selectively in osteocytes (Fig 1C). However, transgene expression was below endogenous *mPit1* mRNA levels in primary bone cells (PBCs) obtained from these mice (S1A Fig) and serum Pi, urine Pi/urine creatinine, and phosphate excretion index (PEI) at P80 were unchanged compared to WT littermates (S1C and S1D Fig). PBC cultures treated with adenovirus encoding cre-recombinase to excise the *LSL*-cassette *in vitro* and to activate the CAG promotor showed several orders of magnitude higher expression of *HA-hPIT1* when compared to untreated *H11-CAG-LSL-HA-hPIT1*^*tg*^ *PBC* (S1B Fig).

Since female transmission of *Dmp1-cre* permits excision of the *LSL* cassette in the germline, we generated a mouse line with expression of the transgene in all mouse tissues. Recombination was confirmed using specific primers in tail genomic DNA (Fig 1D). Heterozygous *HA-hPIT1*^*tg/+*^ mice were born at the expected Mendelian ratio (Table 1).

**Table 1:** *HA-hPIT1*^*tg/+*^ mice are born at expected Mendelian rate.

Genotype	Expected males	Obtained males	Expected females	Obtained females	Indeterminate sex
***WT***	25%	28%(24)	25%	22%(17)	n/a
***HA-hPIT1***^***tg/+***^	25%	18%(15)	25%	11%(8)	n/a
**Dead/ungenotyped/ missing pups**		4.6%(4)		3.5%(3)	7%(6)

Number of mice is shown in brackets (n)

### Expression of *HA-hPIT1* is 10-fold above *mPit1* in *HA-hPIT1*^*tg/+*^ primary calvaria osteoblasts (PCOB)

**Fig 2:**
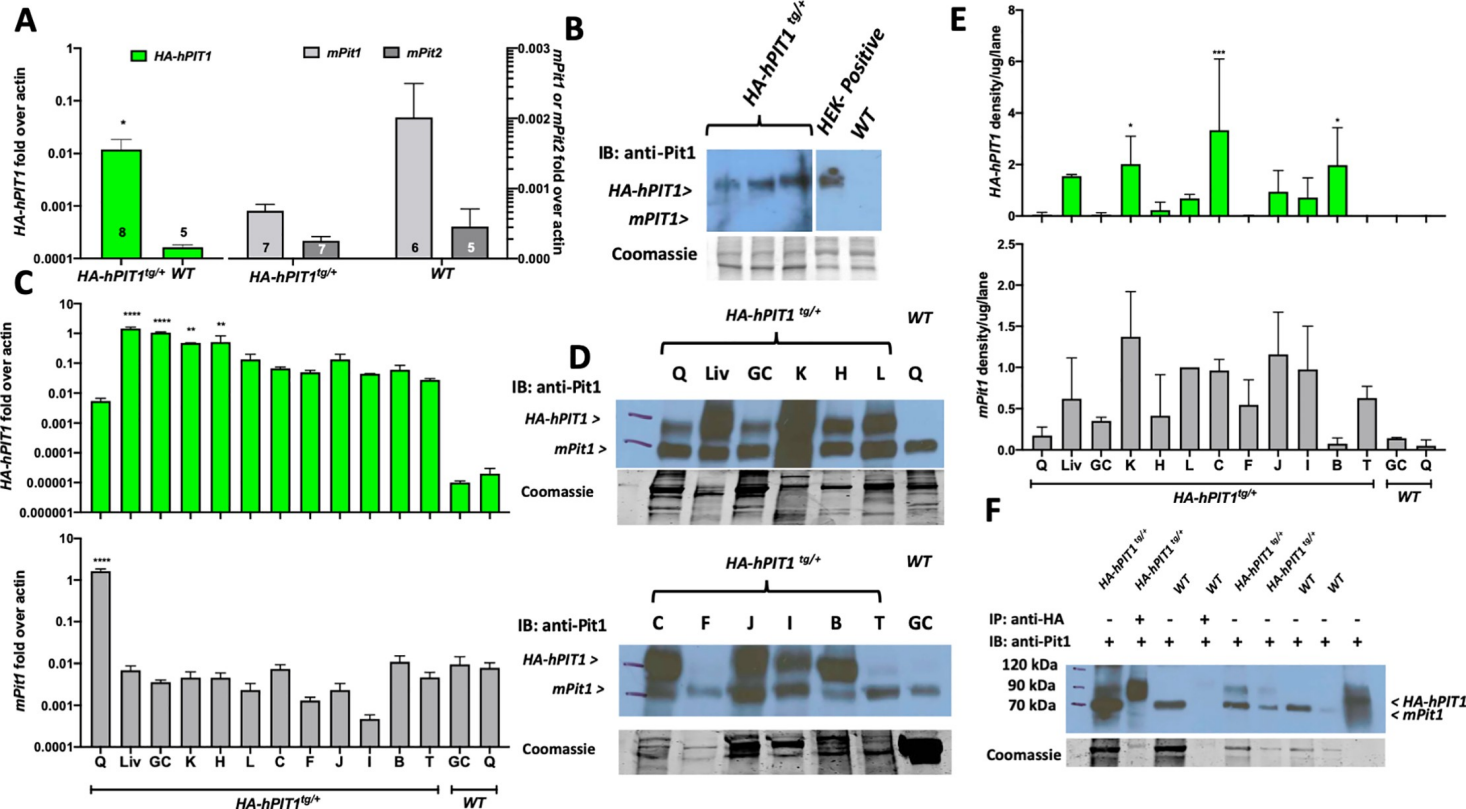
Expression of *HA-hPIT1* and *mPit1* in *HA-hPIT1*^*tg/+*^ *and WT* primary calvaria osteoblasts and various tissues. (A) Semi-quantitative RT-PCR showing fold over actin expression in primary calvaria osteoblasts compared to wildtype at 80 days for *hPIT1* and endogenous mouse *Pit1 and 2 (mPit1 and 2)*. (B) Western blot of lysates obtained from PCOB from three separate *HA-hPIT1*^*tg/+*^ and one *WT* mouse using a lysate of HEK293 cells transfected with an expression plasmid encoding HA-hPIT1 as positive control, hybridized with anti-PIT1 rabbit polyclonal antibody (H-130, sc-98814, Santa Cruz Biotechnology) and detected with horseradish-peroxidase conjugated anti-rabbit IgG (mPit1 expected size 74 kDa, HA-hPIT1 expected size 75 kDa). (C) Semi-quantitative RT-PCR of total RNA from a *HA-hPIT1*^*tg/+*^ mouse shows expression of *hPIT1* in different tissues (Liv = liver, K = kidney, C = colon, J = jejunum, I = ileum, B = brain, L = lung, T = testicle), compared to gastrocnemius (GC) and quadriceps (Q) from a wildtype (*WT*) mouse (n = 2) (D) Western blot of lysates from different tissues of a *HA-hPIT1*^*tg/+*^ and *WT* (hybridized with anti-PIT1 rabbit polyclonal antibody (H-130, sc-98814, Santa Cruz Biotechnology) and detected with horseradish-peroxidase conjugated anti-rabbit IgG) (mPit1 expected size 74 kDa, HA-hPIT1 expected size 75 kDa), (E) densitometric quantification of *hPIT1* and *mPit1* protein normalized to lung *mPit1* and protein per lane from the Coomassie stains from two replicate Western blot experiments. (F) immunoprecipitation of *HA-hPIT* from quadriceps muscle obtained from a WT and *HA-hPIT1*^*tg/+*^ mouse. ****p<0.00002, ***p = 0.0002, **p = 0.002, *p = 0.03 vs. WT.

Different from *HA-hPIT1*^*Dmp1-CO;tg /+*^ mice, germline transgenic mice showed approximately 10-fold higher expression of *HA-hPIT1* when compared to endogenous *mPit1* in PCOBs prepared from P80 mice (0.01+/-0.006 vs 0.0007+/-0.0001, n = 7–8, p = 0.01) (Fig 2A). Expression of endogenous *mPit1* was furthermore slightly, albeit non-significantly, suppressed in *HA-hPIT1*^*tg/+*^ PCOB while *mPit2* was unchanged (Fig 2A). Similarly, an immunoblot of lysates obtained from PCOB of *HA-hPIT1*^*tg/+*^ mice showed expression of *HA-hPIT1*, while endogenous *mPit1* is not detected (Fig 2B).

### Expression of HA-hPIT1 varies between mouse tissues

Next, we prepared total RNA and protein lysates from various mouse tissues at P80. Semiquantitative evaluation by qRT-PCR shows that the *HA-hPIT1* transcript is detected above endogenous *mPit1* in gastrocnemius muscle (1.1+/-0.05 vs 0.004+/-0.0004, n = 2, p<0.0001), but interestingly not in quadriceps muscle (0.005+/-0.001 vs 1.6+/-0.2, n = 2, p = ns), while all other organs show two orders of magnitude higher expression of *HA-hPIT1* compared to endogenous *mPit1* (Fig 2C). However, there is considerable variability between organs, which is found as well in immunoblot analysis which shows protein expression is highest in liver, kidneys, colon, and brain (Fig 2D and 2E). Presence of the HA-tag in the transgene permits immunoprecipitation using HA-agarose as shown for quadriceps muscle (Fig 2F).

### Effect of *HA-hPIT1* transgenic expression on biochemical, cortical and trabecular bone parameters and tissue histology

**Fig 3:**
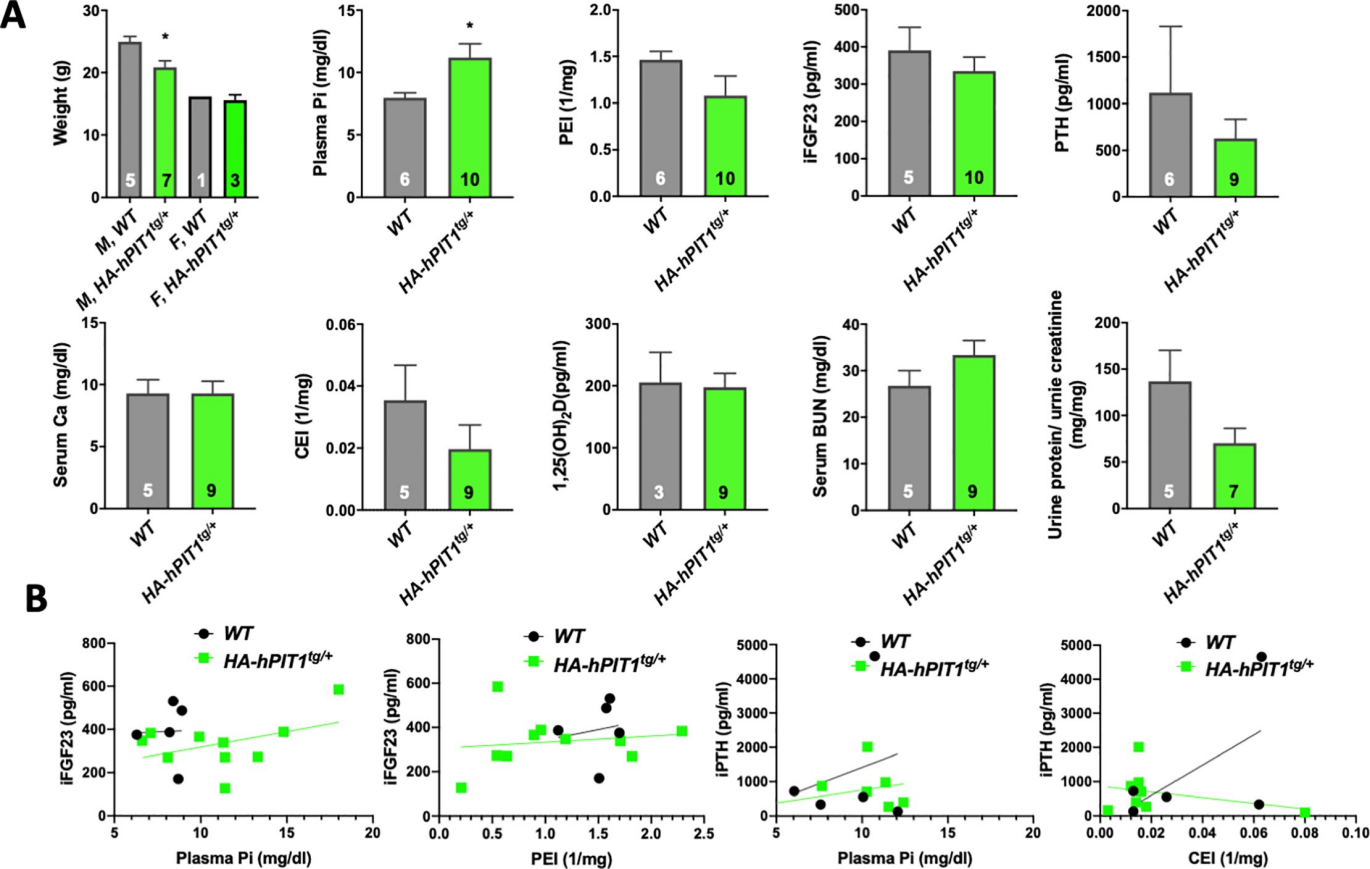
Effect of *HA-hPIT1* overexpression on biochemical parameters. Biochemical parameters were determined in 80 day old mice and show aside from hyperphosphatemia no significant differences of means (**A**) and linear regression analysis (**B**) between WT and *HA-hPIT1*^*tg/+*^ mice. Means±SEM, n indicated by numbers inside bars, *p = 0.03 vs. WT.

**Fig 4:**
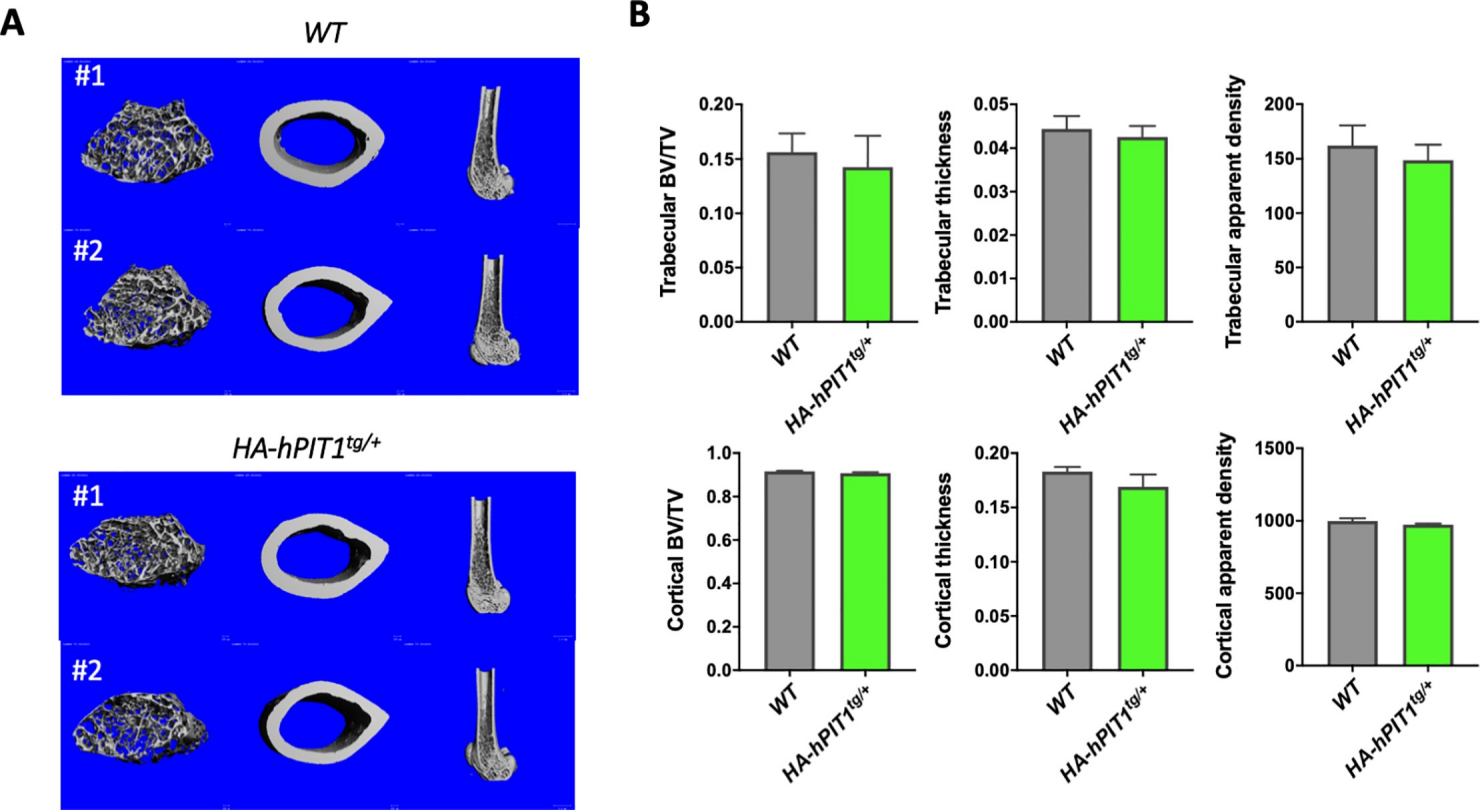
uCT analysis of P80 *HA-hPIT1* transgenic mice. Representative μCT images of two WT and *HA-hPIT1*^*tg/+*^ 80 day old males (A) and quantification of trabecular and cortical parameters (B) shows no significant effect of HA-hPIT1 overexpression. Means±SEM, n = 4 mice.

Phenotypic evaluation showed a small but significant weight reduction of male *HA-hPIT1*^*tg/+*^ mice when compared to WT at 80 days, while we observed no weight difference among genotypes for females (Fig 3A). Transgenic mice were hyperphosphatemic, while there was no significant difference for plasma iPTH, iFGF23, serum calcium, BUN, and 1,25-dihydroxy vitamin D levels, and urine phosphate, calcium, and protein excretion (Fig 3). Likewise, cortical BV/TV and trabecular BV/TV and other parameters obtained in uCT analysis of femurs from *HA-hPIT1*^*tg/+*^ mice at P80 were not significantly different when compared to WT (Fig 4). Furthermore, no differences in tissue mineralization were observed in a tissue histological survey between *HA-hPIT1*^*tg/+*^ at 80 days compared to WT. Finally, using transmission electron microscopic evaluation, glomerular podocytes and glomerular basal membranes were indistinguishable (S2 Fig) [21].

## Discussion

In contrast to traditional Rosa26 knockins, TARGATT allows a large transgene to be integrated into a “safe” locus such as *H11* by simple pronuclear injection. Although not as efficient as initially described [16], this approach avoids the time-consuming generation of ES cells, which permitted generation of conditional offspring within three months. Furthermore, unpredictable genomic events which are a common problem due to multiple insertions of plasmids using standard transgenesis or off-target recombination using CRISPR/Cas9 technology can be detected in the TARGATT approach using integration-site specific primers.

Unfortunately, the 8 kb-*Dmp1-Cre* mice did not yield sufficiently high expression of the transgene in PCOBs obtained from *HA-hPIT1*^*Dmp1-CO;tg/+*^ mice, which is most likely due to inefficient activity of this particular *Dmp1-Cre*, since we were able to obtain robust expression of the transgene after adenoviral transduction of *Cre* in PCOBs *in vitro*. Thus, we decided to first focus this initial phenotypic evaluation on heterozygous *HA-hPIT1*^*tg/+*^mice.

*HA-hPIT1*^*tg/+*^ mice were born at Mendelian ratio and showed transgene expression in PCOB and various mouse tissues up to 100-fold above endogenous *mPit1*. Moreover, protein expression of the transgene was significantly higher than endogenous *mPit1* expression in gastrocnemius muscle, liver, kidneys, colon, and brain based on semi-quantitative RT-PCR, which was corroborated in Western blot analysis. Presence of the HA-tag furthermore permitted immunoprecipitation using quadriceps muscle lysates and anti-HA agarose. These findings indicate that our mice may be a useful tool to study the biology of *hPIT1* and its binding partners that mediate ERK1/2 activation. Since protein expression is more variable than mRNA expression, our data may suggest that posttranscriptional processing and protein stability of *HA-hPIT1* may underly tissue specific regulation, which is of great interest in light of emerging information that type III transporters are not only expressed in the cell membrane, but also in the endoplasmic reticulum [22] and that they have transport-independent functions which include regulation of insulin sensitivity and of cell proliferation [9, 23].

Supraphysiological expression of *hPIT1* may affect matrix mineralization. However, bone mineralization appeared to be normal following uCT analysis of *HA-hPIT1*^*tg/+*^ mice. This is consistent with absence of a bone and mineral metabolism phenotype in *Pit1* hypomorphic mice [12]. However, further evaluation, for example of the time-course and response to stressors such as a high phosphate, high fat diet or in a cross with *Pit2*, *Phospho1*, and *Tnsalp* null mice, may be of interest to see whether overexpression of *HA-hPIT1* affects vascular calcification or matrix vesicles mineralization and bone quality defects observed in these mice [3, 24, 25].

We hypothesized that supraphysiological expression of *hPIT1* in parathyroids, osteocytes, intestine, and proximal tubules of the kidneys may affect mineral metabolism. P80 *HA-hPIT1*^*tg/+*^ mice indeed were hyperphosphatemic as was observed by Suzuki et al. in *Pit1* transgenic rats [13]. However, we were unable to detect the hyperparathyroidism, or bone changes consistent with osteomalacia or osteoporosis reported by these authors, which may be due to species differences or insertion site related effects, since the rat-transgene is randomly inserted. Likewise, GBM thickening was absent in our transgenic mice, although it was reported in the transgenic rats [21].

The hyperphosphatemia in our *HA-hPIT1*^*tg/+*^ mice is likely due to hyperabsorption of Pi from the diet in the gut and in the proximal tubules of the kidneys, but may be compensated by increased cellular uptake of Pi into peripheral tissues. Compensation by down-regulation of endogenous *mPit1* in osteocytes, parathyroids, and proximal tubule cells may furthermore explain why we were unable to detect changes in blood intact FGF23, intact PTH, and 1,25(OH)_2_-vitamin D levels, although it is also possible that regulation of hormone secretion by Pi is more complex than previously thought [1, 3, 26–28]. Compensatory expression of renal phosphate transporters, including the type II sodium-phosphate cotransporters *Npt2a/c*, may contribute to why we were unable to detect changes in urinary phosphate, which may warrant further study using brush border membrane preparation or immunohistochemistry of kidney sections. Interpretation of these results may be difficult, since overexpression of *HA-hPIT1* in the parathyroids or osteocytes may cause systemic effects in addition to regulation of these transporters in a cell-autonomous fashion. In our view, questions of compensatory regulation therefore are better addressed using conditional transgenic or *in vitro* approaches. Our conditional transgene will be a useful tool for evaluation of these tissue specific metabolic and endocrine functions of *hPIT1* in bone and mineral metabolism.

In summary, we used the TARGATT technology to insert a C-terminal *HA-epitope tagged hPIT1* transgene under the control of the CAG promotor into a modified *H11* locus. Initial characterization shows that heterozygous expression of *HA-hPIT1* in the germline is compatible with life and causes hyperphosphatemia, while bone and mineral metabolism of these mice is otherwise normal.

## Supporting information

S1 Fig *HA-hPIT1*^*Dmp1-CO;tg /+*^mice lack transgene expression in primary bone cells and have normal plasma Pi, urine Pi/urine creatinine and PEI. Semi-quantitative qRT-PCR of hPIT1 and mPit1 expression in primary bone cells (**A**), in which *HA-hPIT1* expression is low despite *Dmp1-Cre*, but can be induced by treatment with Adeno-Cre *in vitro* (**B**) (n = 4). Serum Pi (**C**) urine Pi/urine creatinine (**D**) and PEI (**E**) of *HA-PIT1*^*Dmp1-CO;tg /+*^mice is unchanged compared to WT littermates at P80. Means±SEM, n = 15 mice.
(DOCX)

S2 Fig TEM of *HA-hPIT1*^*tg/+*^ *shows no apparent podocyte injury and GBM thickening*. Following fixation and epoxy embedding uranium and lead stained sections from WT and HA-hPIT1tg/+ mice were observed using a Tecnai Biotwin (LaB6, 80 kV) (FEI, Thermo Fisher, Hillsboro, OR). ‘>‘ indicate the glomerular basal membrane (GBM)
(DOCX)

S1 Raw Images (PDF)

S1 Table Primer sequences. *F = forward, R = reverse.
(DOCX)
