# Supplementary material for: Transgenic mouse model for conditional expression of influenza hemagglutinin-tagged human SLC20A1/PIT1
Source: PLoS One. 2019 Oct 15;14(10):e0223052. doi: 10.1371/journal.pone.0223052 (PMC6793878; doi:10.1371/journal.pone.0223052)
Supplement: S1 Fig — Semi-quantitative qRT-PCR of hPIT1 and mPit1 expression in primary bone cells (A), in which HA-hPIT1 expression is low despite Dmp1-Cre, but can be induced by treatment with Adeno-Cre in vitro (B) (n = 4). Serum Pi (C) urine Pi/urine creatinine (D) and PEI (E) of HA-PIT1Dmp1-CO;tg /+mice is unchanged compared to WT littermates at P80. Means±SEM, n = 15 mice. (DOCX) [file pone.0223052.s001.docx]

**Supporting information for : Transgenic mouse model for conditional expression of** **influenza hemagglutinin-tagged human *SLC20A1/PIT1***

Sampada Chande, Bryan Ho, Jonathan Fetene, Clemens Bergwitz


#

#


# S1 Fig: *HA-hPIT1^Dmp1-CO;tg /+^*mice lack transgene expression in primary bone cells and have normal plasma Pi, urine Pi/urine creatinine and PEI.

# Semi-quantitative qRT-PCR of hPIT1 and mPit1 expression in primary bone cells (A), in which *HA-hPIT1* expression is low despite *Dmp1-Cre,* but can be induced by treatment with Adeno-Cre *in vitro* (B) (n=4). Serum Pi (C) urine Pi/urine creatinine (D) and PEI (E) of *HA-PIT1^Dmp1-CO;tg /+^*mice is unchanged compared to WT littermates at P80. Means±SEM, n=15 mice.
