# Supplementary material for: Transgenic mouse model for conditional expression of influenza hemagglutinin-tagged human SLC20A1/PIT1
Source: PLoS One. 2019 Oct 15;14(10):e0223052. doi: 10.1371/journal.pone.0223052 (PMC6793878; doi:10.1371/journal.pone.0223052)
Supplement: S2 Fig — Following fixation and epoxy embedding uranium and lead stained sections from WT and HA-hPIT1tg/+ mice were observed using a Tecnai Biotwin (LaB6, 80 kV) (FEI, Thermo Fisher, Hillsboro, OR). ‘>‘ indicate the glomerular basal membrane (GBM) (DOCX) [file pone.0223052.s002.docx]

**Supporting information for : Transgenic mouse model for conditional expression of** **influenza hemagglutinin-tagged human *SLC20A1/PIT1***

Sampada Chande, Bryan Ho, Jonathan Fetene, Clemens Bergwitz


#

#


# S2 Fig: TEM of *HA-hPIT1^tg/+^ shows no apparent podocyte injury and GBM thickening*

# Following fixation and epoxy embedding uranium and lead stained sections from WT and HA-hPIT1tg/+ mice were observed using a Tecnai Biotwin (LaB6, 80 kV) (FEI, Thermo Fisher, Hillsboro, OR). ‘>’ indicate the glomerular basal membrane (GBM)
