# Supplementary material for: Transgenic mouse model for conditional expression of influenza hemagglutinin-tagged human SLC20A1/PIT1
Source: PLoS One. 2019 Oct 15;14(10):e0223052. doi: 10.1371/journal.pone.0223052 (PMC6793878; doi:10.1371/journal.pone.0223052)

Method to capture image: Quantity one software Biorad

Figure panel: Figure 1(B)

Recombined *H11* allele- at 3’ junction (2,4)

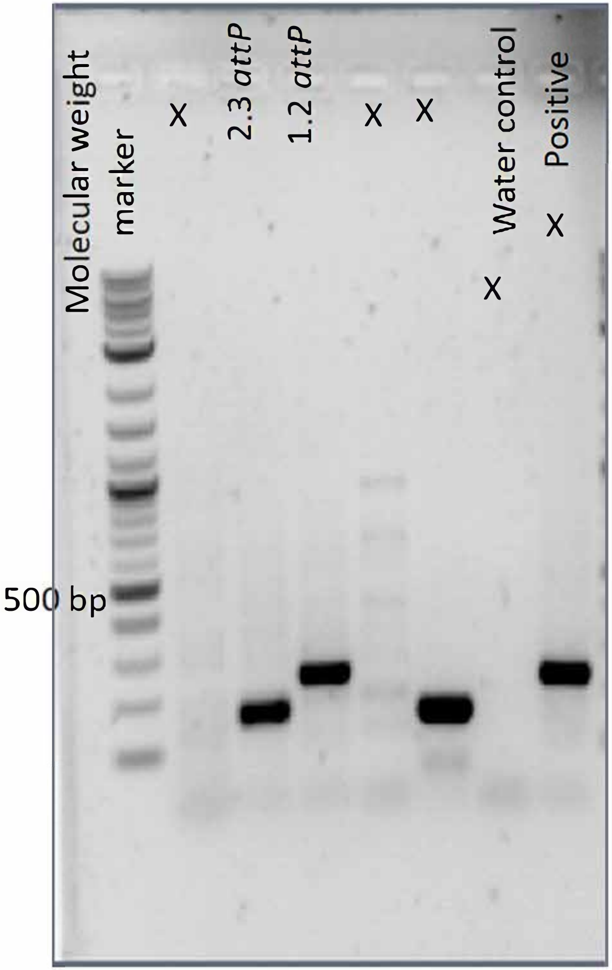

Recombined *H11* allele- at 5’ junction (1,3)

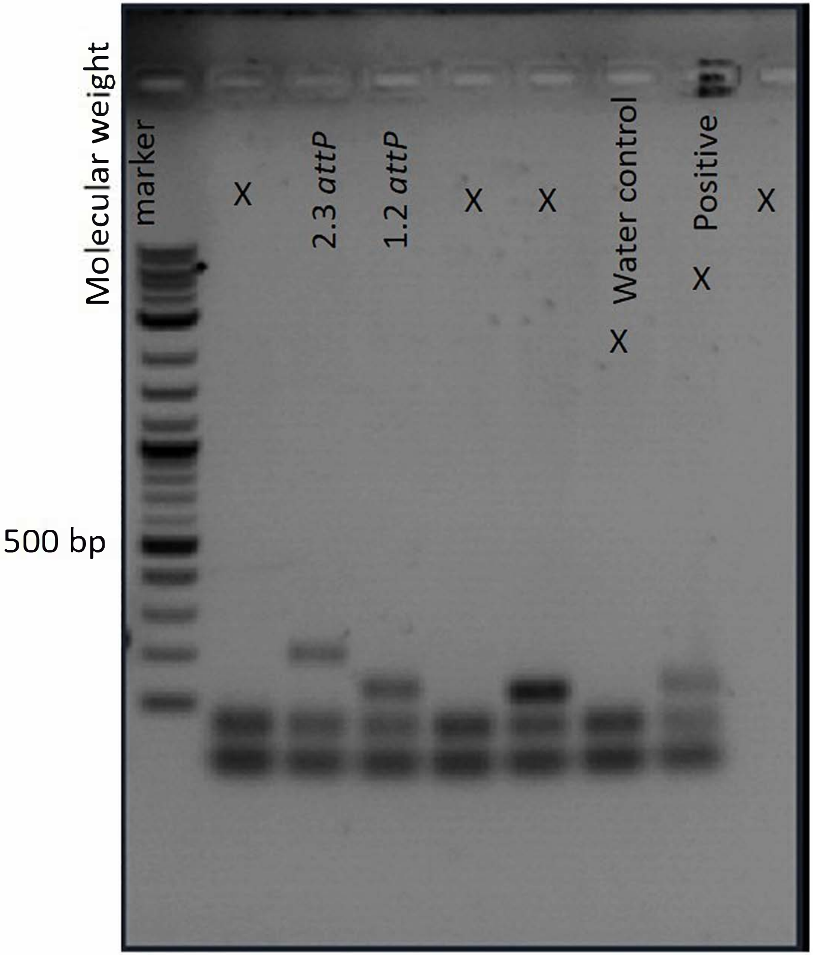

Method to capture image: Quantity one software Biorad

Figure panel: Figure 1(D)

H11 allele (896,897) at 356 bp

CAG-LSL-HA-hPIT1 (879,883) at 530 bp

Cre-recombinase (823,824) at 400 bp

gΔLSL (880, 883) at 450 bp

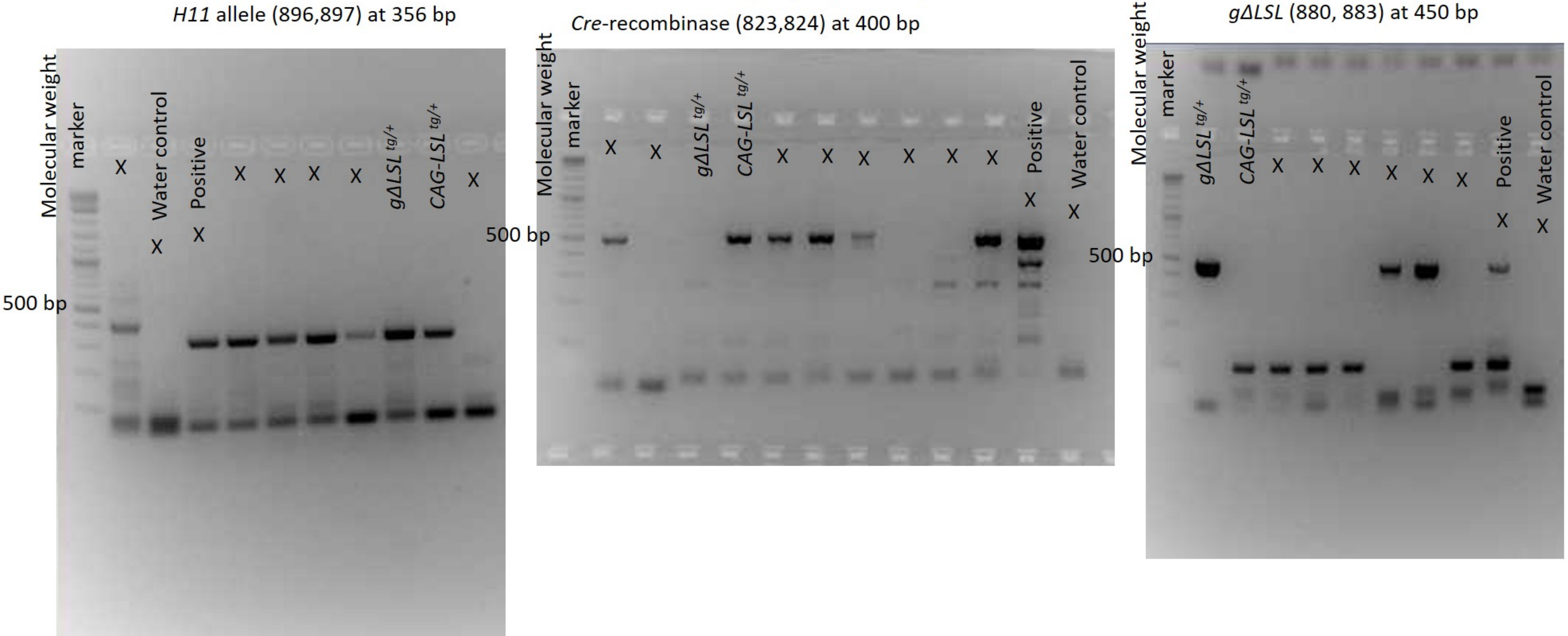

Method to capture image: Scanned autoradiography film
Figure panel: Figure 2(B)

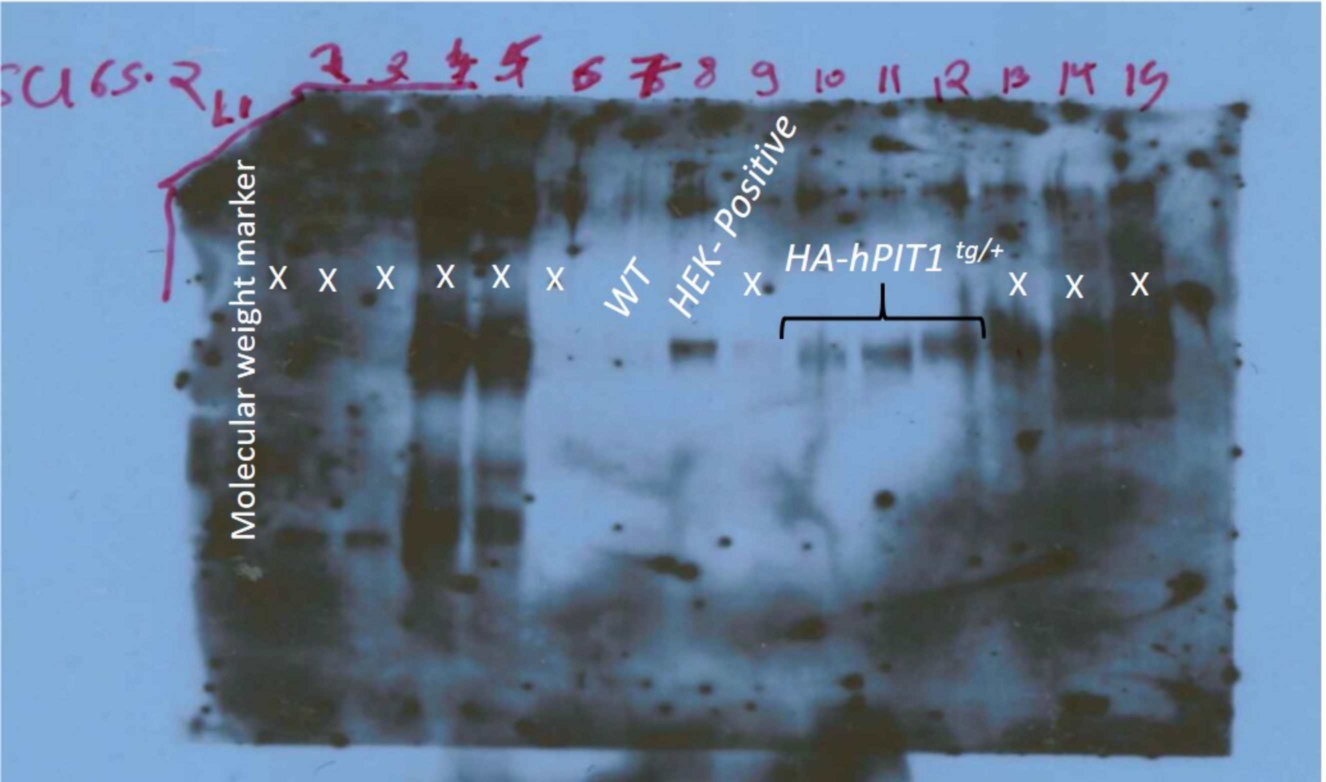

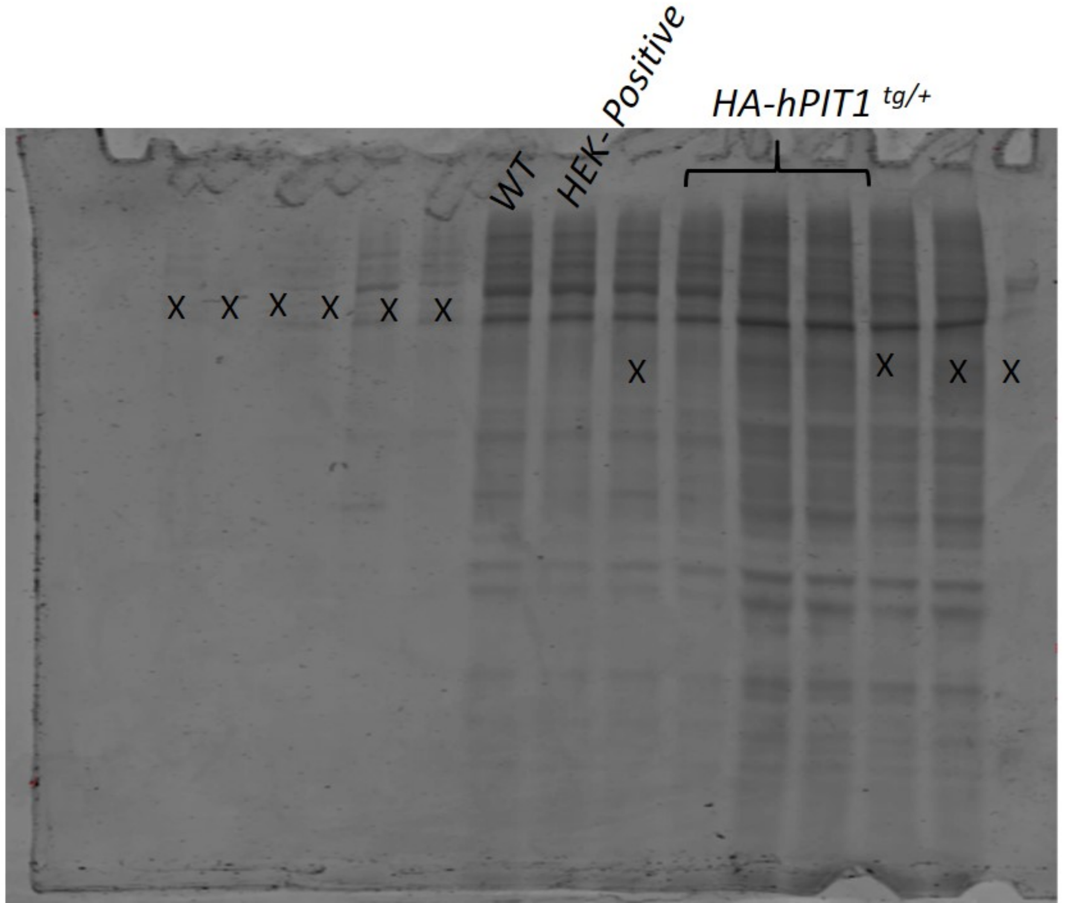

Method to capture image: Scanned autoradiography film
Figure panel: Figure 2(D)

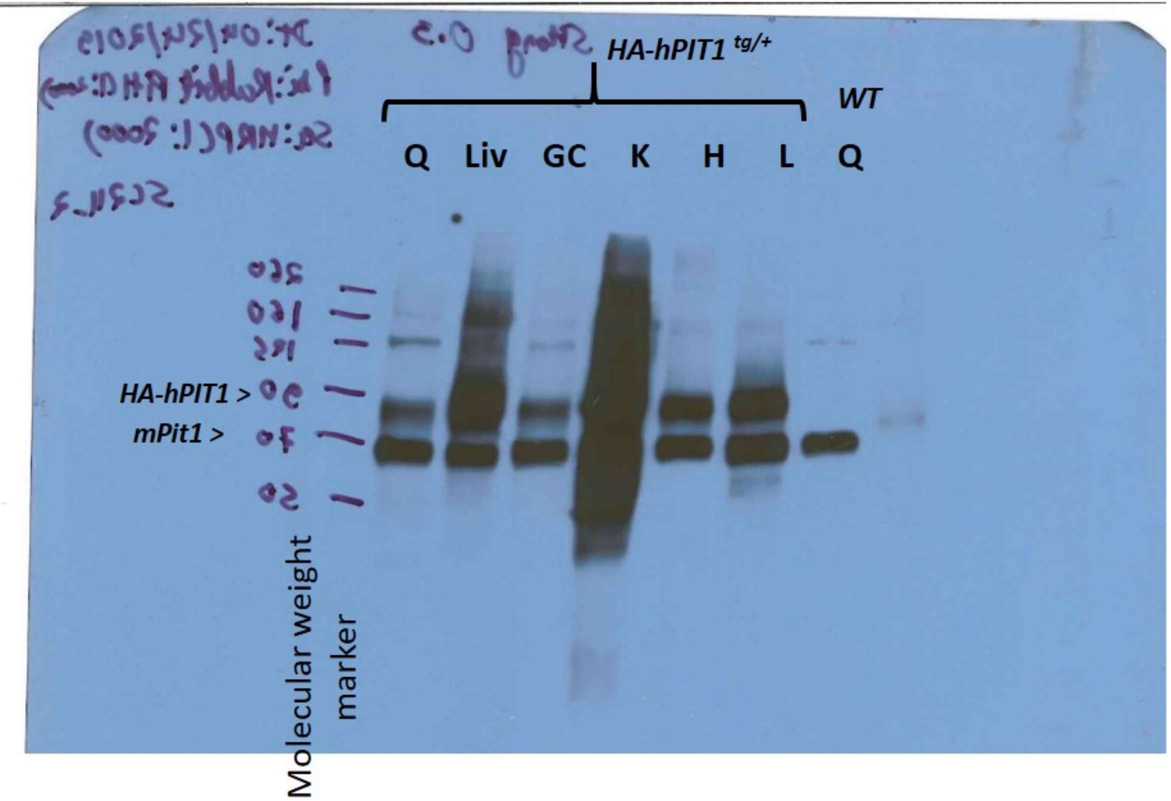

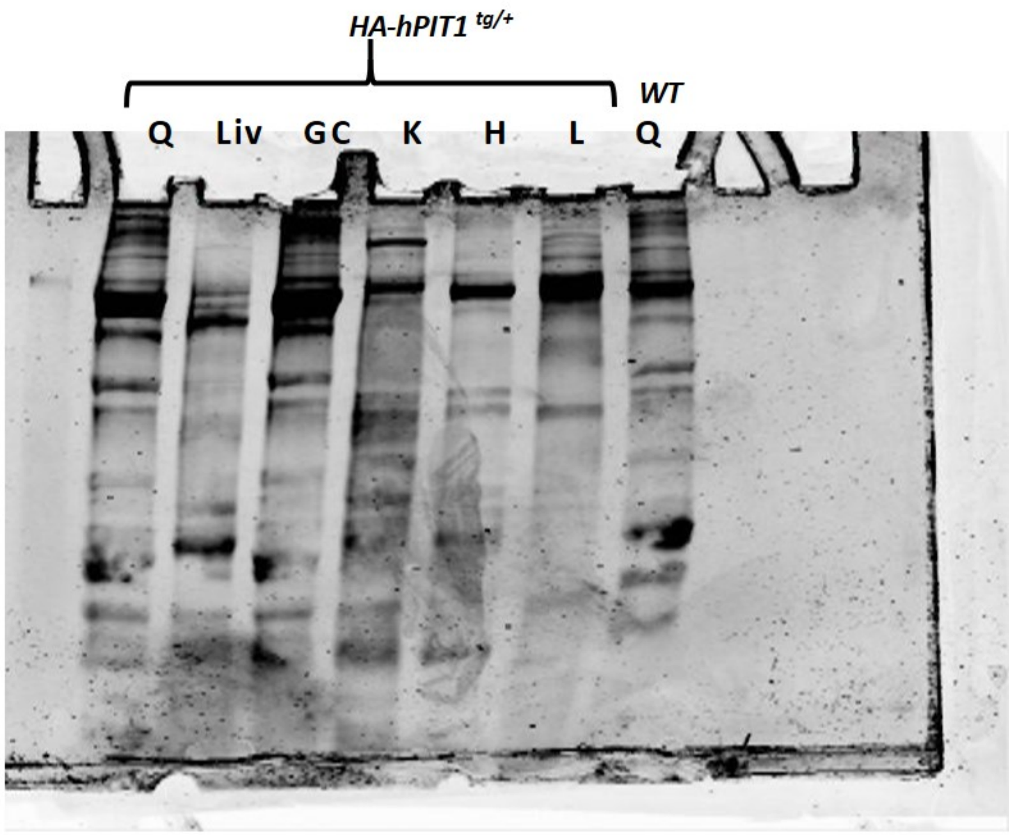

Method to capture image: Scanned autoradiography film

Figure panel: Figure 2(D)

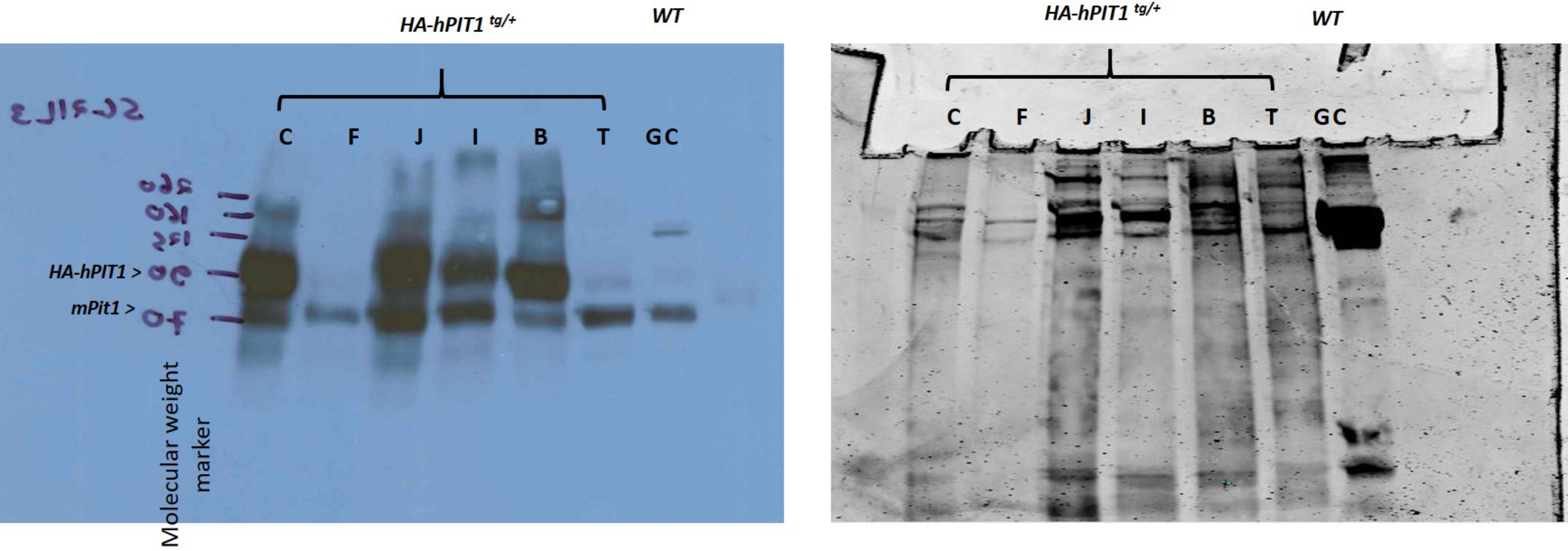

Method to capture image: Scanned autoradiography film

Figure panel: Figure 2(F)

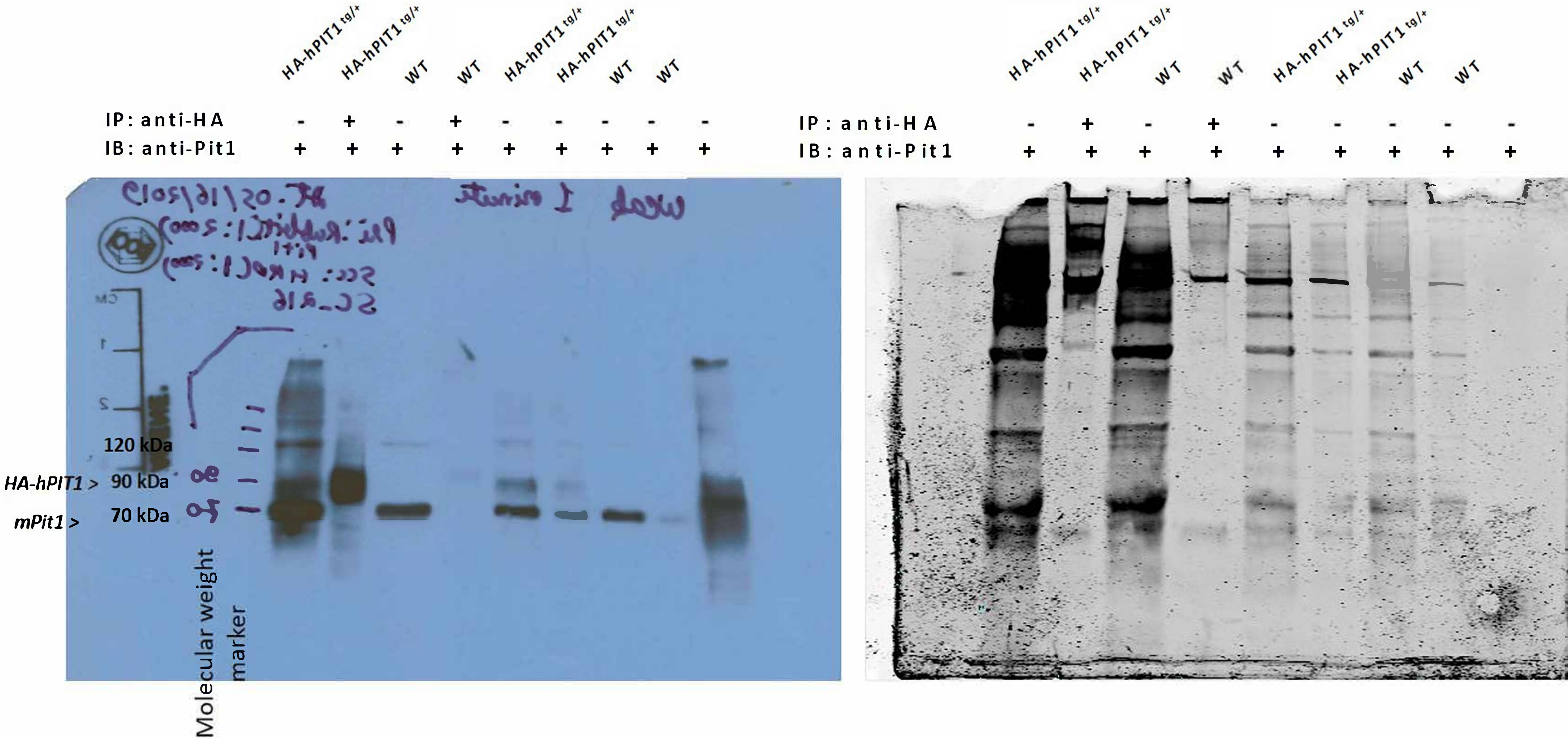

Supplement: S1 Raw Images — (PDF) [file pone.0223052.s003.pdf]
