# Supplementary material for: Transgenic mouse model for conditional expression of influenza hemagglutinin-tagged human SLC20A1/PIT1
Source: PLoS One. 2019 Oct 15;14(10):e0223052. doi: 10.1371/journal.pone.0223052 (PMC6793878; doi:10.1371/journal.pone.0223052)
Supplement: S1 Table — *F = forward, R = reverse. (DOCX) [file pone.0223052.s004.docx]

**Supporting information for : Transgenic mouse model for conditional expression of** **influenza hemagglutinin-tagged human *SLC20A1/PIT1***

Sampada Chande, Bryan Ho, Jonathan Fetene, Clemens Bergwitz


#

#

# S1 Table: Primer sequences

| ID | Gene | * | sequence |
| --- | --- | --- | --- |
| 1 | SSL | F | Applied StemCell, Inc., Milpitas, CA (proprietary sequence |
| 3 | SSL | R | Applied StemCell, Inc., Milpitas, CA (proprietary sequence |
| 4 | SSR | F | Applied StemCell, Inc., Milpitas, CA (proprietary sequence |
| 2 | SSR | R | Applied StemCell, Inc., Milpitas, CA (proprietary sequence |
| 179 | *mß-actin* | F | GGCTGTATTCCCCTCCATCG |
| 180 | *mß-actin* | R | CCAGTTGGTAACAATGCCATGT |
| 816 | mutagenesis | F | GCACCCGGGCGCGCCaccATGGCAACGCTGATTACCAG |
| 817 | mutagenesis | R | GCAGCGCGCAAGCTTCAagcgtaatctggaacatcgtatgggtaCATTCTGAGGATGACATAT |
| 823 | *CreY-1 F* | F | TGCCACGACCAAGTGACAGCAATG |
| 824 | *CreY-1 R* | R | AGAGACGGAAATCCATCGCTCG |
| 838 | *mPit1* | F | TGTATTGTCGGTGCAACCAT |
| 839 | *mPit1* | R | ATACCAGAAAGCAGCGGAGA |
| 841 | *mPit2* | F | ACCATTCGGAAAGGCATCATT |
| 842 | *mPit2* | R | GAAGGACGCGATCAACTGC |
| 879 | *LSL* | F | CACTGCATTCTAGTTGTGGTTTGTCC |
| 880 | *LSL* | R | GAGCCCACTGTTTCAAAGATGCTAG |
| 883 | *ΔLSL* | F | GCAAAGAATTCGCGCGTCTT |
| 890 | *hPIT1* | F | GGAAGGGCTTGATTGACGTG |
| 891 | *hPIT1* | R | CAGAACCAAACATAGCACTGACT |
| 896 | *H11 locus* | F | ACTCTACTGGAGGAGGACAAACTGGTCAC |
| 897 | *H11 locus* | R | TTGTTCCCTTTCTGCTTCATCTTGCTGA |

# *F=forward, R=reverse
